# Impact of thyroid hormone replacement on the risk of second cancer after thyroidectomy: a Korean National Cohort Study

**DOI:** 10.1038/s41598-023-43461-8

**Published:** 2023-09-28

**Authors:** Joon Ho, Minkyung Han, Inkyung Jung, Young Suk Jo, Jandee Lee

**Affiliations:** 1 Department of Surgery, Open NBI Convergence Technology Research Laboratory, Yonsei University College of Medicine, Seoul, South Korea; 2 Biostatistics Collaboration Unit, Department of Biomedical Systems Informatics, Yonsei University College of Medicine, Seoul, South Korea; 3 Division of Biostatistics, Department of Biomedical Systems Informatics, Yonsei University College of Medicine, Seoul, South Korea; 4 Department of Internal Medicine, Yonsei University College of Medicine, Seoul, South Korea

**Keywords:** Endocrine cancer, Thyroid diseases, Cancer prevention

## Abstract

We aimed to investigate the effect of thyroid hormone administration on the risk of second primary cancer in patients who underwent thyroidectomy for differentiated thyroid cancer. Data were extracted from the medical billing data of the Health Insurance Review and Assessment Service in South Korea. Patients between 19 and 80 years old who underwent thyroid surgery at least once between January 2009 and June 2020 were included. Data of patients with second primary cancer and control patients with matched age, sex, operation date, and follow-up duration were extracted at a ratio of 1:4. A nested case–control analysis was performed to exclude length bias to confirm the correlation between the duration of thyroid hormone administration, dose, and incidence of second primary cancer. Of the 261,598 patients who underwent surgery for thyroid cancer included in the study, 11,790 with second primary cancer and 47,160 without second primary cancer were matched. The average dose of thyroid hormone increased the adjusted odds ratio (OR) for both low (≤ 50 μg, OR 1.29, confidence interval (CI) 1.12–1.48) and high (< 100 μg, OR 1.24, CI 1.12–1.37) doses. Analyzing over time, the adjusted OR of second primary cancer increased, especially in short (≤ 1 year) (OR 1.19; CI 1.06–1.34) and long (> 5 years) duration (OR 1.25; CI 1.10–1.41). In conclusion, insufficient and excessive thyroid hormone replacement might be linked to increased second primary cancer in patients who underwent thyroidectomy for differentiated thyroid cancer.

## Introduction

Thyroid cancer is the most common endocrine malignancy, and its incidence has been increasing worldwide^1–5^. Most patients experience long-term survival owing to favorable prognosis of differentiated thyroid cancer (DTC). Because of an excellent prognosis, managing survivors after diagnosing and treating patients with DTC has been an important issue. Although DTC shows favorable clinical outcomes, tumor progression or recurrence remains a concern. Another concern is treatment-related or unrelated secondary primary malignancy. Treatment-related secondary cancers such as leukemia and lymphoma have been reported in patients with DTC who received radioactive iodine therapy^6–8^. In addition, as obesity has been regarded as a risk factor for DTC, the increased incidence of obesity-related cancers, such as breast and colon cancers, has also been reported in survivors of DTC^9–12^.

Many researchers and physicians are interested in various cardiovascular and metabolic risks caused by long-term thyroid hormone replacement (THR) after thyroidectomy^13–15^. Thyroid dysfunction, including hyperthyroidism and hypothyroidism, has also been investigated as a risk or preventive factor for human cancers. Yuan et al. reported that thyroid dysfunction might increase the overall cancer incidence and the risk of breast cancer^16^. Boursi et al. reported that thyroid dysfunction is associated with an increased risk of colorectal cancer; however, long-term THR has a protective effect^17^. In contrast, a large-scale population-based study in Sweden reported that levothyroxine treatment increased the risk of overall cancer^18^. To suppress thyrotropin, patients with DTC who underwent thyroidectomy had taken THR for a considerable period of time determined by surgical methods and recurrence risk^19–23^. Therefore, THR might be associated with the risk of second primary cancer in these patients.

This study aimed to determine the risk of second primary cancer according to the duration and dose of thyroid hormone administration in patients undergoing thyroidectomy for DTC. For a large-scale cohort study targeting the entire population, big data from the Health Insurance Review and Assessment Service (HIRA) was used.

## Results

### Study summary and baseline characteristics

**Figure 1:**
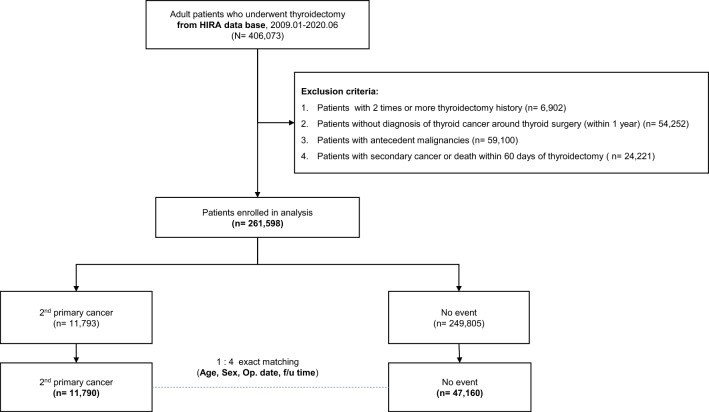
Schematic flow of study design using Korean national cohort. *HIRA* health insurance review and assessment service.

Data of patients who underwent thyroid surgery during the study period were extracted from the HIRA database. In total, 406,073 patients were included in the analysis. The data of 261,598 patients who underwent surgery for thyroid cancer were extracted after considering the exclusion criteria. Of these patients, 11,790 with second primary cancer and 47,160 without second primary cancer (1:4) were matched for age, sex, operation date, and follow-up duration (Fig. 1). The average age of both groups was 53.1 years old, and 80.47% of the patients were women. The hospital surgery scale, hypertension, diabetes mellitus (DM), dyslipidemia, and history of various cancer-related infections showed statistically significant differences between the two groups (Table 1).

**Table 1:** Baseline characteristics in patients with thyroidectomy history according to secondary cancer in Korean national cohort after matching.

	Second primary cancer		P-value
	Absence (n = 47,160)	Presence (n = 11,790)	
Age at surgery, mean ± SD	53.1 ± 11.4	53.1 ± 11.5	
≤ 40	5800 (12.30)	1450 (12.30)	
40 < and ≤ 60	27,228 (57.74)	6807 (57.74)	
60 <	14,132 (29.97)	3533 (29.97)	
Sex, n (%)			
Men	9212 (19.53)	2303 (19.53)	
Women	37,948 (80.47)	9487 (80.47)	
Hospital scale^a^ at surgery, n (%)			< 0.001
Tertiary	30,461 (64.59)	7261 (61.59)	
General	14,209 (30.13)	3886 (32.96)	
Community	1617 (3.43)	452 (3.83)	
Clinics	873 (1.85)	191 (1.62)	
Obesity, n (%)	84 (0.18)	21 (0.18)	1.000
Hypertension, n (%)	16,484 (34.95)	4450 (37.74)	< 0.001
Diabetes mellitus, n (%)	8671 (18.39)	2370 (20.10)	< 0.001
Dyslipidemia, n (%)	18,203 (38.60)	4675 (39.65)	0.037
Infections^b^, n (%)	2058 (4.36)	728 (6.17)	< 0.001
Duration of levothyroxine, n (%)			
Mean ± SD (days)	1187.0 ± 916.8	1203.0 ± 926.6	0.091
No	2674 (5.67)	580 (4.92)	0.009
≤ 1 year	7357 (15.60)	1877 (15.92)	
1 < and ≤ 5 years	26,049 (55.24)	6493 (55.07)	
5 years <	11,080 (23.49)	2840 (24.09)	
Daily average dose of levothyroxine, n (%)			
Mean ± SD	125.8 ± 56.5	129.9 ± 55.9	< 0.001
No	2674 (5.67)	580 (4.92)	< 0.001
≤ 50 μg	1626 (3.45)	450 (3.82)	
50 μg < and ≤ 100 μg	8489 (18.00)	1898 (16.10)	
100 μg <	34,371 (72.88)	8862 (75.17)	
RAI cumulative dose, n (%)			< 0.001
≤ 30 mCi	35,610 (75.51)	8560 (72.60)	
30 mCi < and ≤ 100 mCi	5717 (12.12)	1425 (12.09)	
100 mCi < and ≤ 150 mCi	4261 (9.04)	1176 (9.97)	
150 mCi <	1572 (3.33)	629 (5.34)	

^a^Classified according to bed size, available department, and location. ^b^Epstein-Barr virus (EBV), hepatitis B and C viruses (HBV and HCV), human immunodeficiency virus (HIV), human papilloma virus (HPV), human T-cell leukemia/lymphoma virus type-1 (HTLV-1), and Helicobacter pylori (*H. pylori*).

We classified the duration of THR into three groups: ≤ 1 year (short-term), 1–5 years (medium-term), and > 5 years (long-term). Although the mean duration of THR was not different between the groups, second primary cancers were frequently observed in patients who received either short- or long-term replacements (P = 0.009, Table 1). We also classified the daily average dose of levothyroxine into three groups: ≤ 50 μg (low dose), 50 μg < and ≤ 100 μg (intermediate dose), and > 100 μg (high dose). The mean daily average dose was higher in patients with second primary cancer (P < 0.001, Table 1). However, low and high doses were more frequently administered to patients with second primary cancer (P < 0.001, Table 1). As expected, the cumulative dose of radioactive iodine (RAI) was also higher in patients with second primary cancers (P < 0.001, Table 1). In subgroup analysis of patients who underwent lobectomy, the baseline characteristics were quite similar to those of the entire cohort, and daily average dose of levothyroxine was associated with the presence of second primary cancers (Supplementary Table 1). However, only 12 out of 10,375 of these patients took long-term medication (> 5 years), as, generally, long-term medication was not needed.

### Multivariable analysis

We observed that the risk of second primary cancer was related to hypertension, DM, dyslipidemia, a history of various cancer-related infections, and the cumulative dose of RAI. In addition, as previously reported, the cumulative dose of RAI is a significant risk factor for second primary cancer in patients with PTC (Table 2).

**Table 2:** Multivariable analysis for the association between cumulative dose of RAI and the risk of second primary cancer.

Cumulated dose of RAIT	2nd cancer		Unadjusted OR (95% CI, P)	Adjusted OR (95% CI, P)
	Absence (n = 47,160), no. (%)	Presence (n = 11,790), no. (%)		
No RAIT	31,272 (66.31)	7500 (63.61)	Ref	Ref
RAIT	15,888 (33.69)	4290 (36.39)	1.15 (1.09–1.20, P < 0.001)	1.12 (1.07–1.18, P < 0.001)
≤ 30 mCi	35,610 (75.51)	8560 (72.60)	Ref	Ref
30 mCi < and ≤ 100 mCi	5717 (12.12)	1425 (12.09)	1.05 (0.98–1.12, P = 0.151)	1.03 (0.96–1.10, P = 0.385)
100 mCi < and ≤ 150 mCi	4261 (9.04)	1176 (9.97)	1.17 (1.09–1.26, P < 0.001)	1.14 (1.06–1.22, P < 0.001)
150 mCi <	1572 (3.33)	629 (5.34)	1.70 (1.54–1.87, P < 0.001)	1.66 (1.50–1.83, P < 0.001)

Adjusted for obesity, hypertension, diabetes mellitus, dyslipidemia, infections. *RAI* Radioactive iodine, *RAIT* Radioactive iodine therapy, *OR* odds ratio, *CI* confidence interval.

Based on these findings, we performed a multivariable analysis adjusted for these factors to reveal the association between the daily average dose and duration of THR for second primary cancer. First, we performed a multivariate analysis adjusted for various factors to reveal the association between the daily average dose of levothyroxine and second primary cancer. Interestingly, in the no-replacement group, the low and high doses showed significantly increased OR (Table 3). In multivariable analysis for patients who underwent lobectomy, low doses significantly increased the risk of second primary cancer (Supplementary Table 2).

**Table 3:** Multivariable analysis for the association between the daily average dose of thyroid hormone and the risk of second primary cancer.

Daily dosage (avg. µg)	2nd cancer		Unadjusted OR (95% CI, P)	Adjusted OR (95% CI, P)
	Absence no. (%)	Presence no. (%)		
No	2674 (5.67)	580 (4.92)	Ref	Ref
≤ 50	1626 (3.45)	450 (3.82)	1.27 (1.11–1.47, P = < 0.001)	1.29 (1.12–1.48, P = 0.001)
50 < and ≤ 100	8489 (18.00)	1898 (16.10)	1.05 (0.95–1.17, P = 0.344)	1.07 (0.96–1.18, P = 0.233)
100 <	34,371 (72.88)	8862 (75.17)	1.26 (1.14–1.40, P = < 0.001)	1.24 (1.12–1.37, P = < 0.001)

Avg.: daily average, adjusted for obesity, hypertension, diabetes mellitus, dyslipidemia, and infections. Cumulative dosage of radioactive iodine; *OR* odds ratio, *CI* confidence interval.

Next, we performed a multivariable analysis adjusted for various factors to reveal the association between the duration of levothyroxine use and second primary cancer. Based on the no-replacement group, all three groups classified by THR duration showed an increased OR for second primary cancer (Table 4). However, a time-dependent increase in the OR was not observed. To understand the risk of individual second primary cancers, we analyzed the OR for the effect of THR duration. As shown in Supplementary Table 1, most crude ORs increased after short- and long-term administration. However, statistical significance was observed in only a small number of second primary cancers, such as liver, lung, bronchial, and brain cancers. Exceptionally, skin cancer and melanoma showed decreased ORs with THR (Supplementary Table 3).

**Table 4:** Impact of the duration of thyroid hormone replacement on the risk of second primary cancer.

Duration	2nd primary cancer		Unadjusted OR (95% CI, P)	Adjusted OR (95% CI, P)
	Absence (n = 47,160), no. (%)	Presence (n = 11,790), no. (%)		
No	2674 (5.67)	580 (4.92)	Ref	Ref
≤ 1 year	7357 (15.60)	1877 (15.92)	1.20 (1.06–1.34, P = 0.003)	1.19 (1.06–1.34, P = 0.003)
1 < and ≤ 5 years	26,049 (55.24)	6493 (55.07)	1.16 (1.05–1.28, P = 0.005)	1.15 (1.04–1.28, P = 0.006)
5 years <	11,080 (23.49)	2840 (24.09)	1.27 (1.12–1.43, P < 0.001)	1.25 (1.10–1.41, P < 0.001)

Adjusted for obesity, hypertension, diabetes mellitus, dyslipidemia, infections, and cumulative dosage of radioactive iodine; *OR* odds ratio, *CI* confidence interval.

## Discussion

In this study, using a large population-based cohort, we observed that the risk of second primary cancer increased according to the duration and dose of levothyroxine administered after thyroidectomy. To exclude lead time bias, a nested case–control study using exact matching was conducted to derive the results. It can be interpreted that the period that affects the occurrence of second primary cancer is shorter in patients who take the drug for a short duration. Furthermore, the period until occurrence is shorter. However, to prevent such misinterpretation, the follow-up period was matched and analyzed in patients with and without second primary cancer. And as mentioned above, it can be confirmed from the result that there was no significant difference in the average duration of levothyroxine administration between the two patient groups.

Several previous studies have reported an increased risk of second primary cancer in patients with thyroid cancer using population-based cohorts. For example, a population-based study in Taiwan analyzed 19,068 patients with thyroid cancer and reported that the risk of second primary cancer increased in both men and women compared with the general population (SIR 1.33, 95% CI 1.23–1.44)^24^. A large European cohort study analyzed 6841 patients with thyroid cancer and reported a 27% increase in the risk of second primary cancer compared to the general population (95% CI 15–40)^14^. This increase in the risk of second primary cancer was analyzed for several reasons. Most representatively, an increase in hematologic malignancy was explained by RAI treatment-related adverse events^8, 25^. In addition, an increase in second primary cancers was associated with the promotion of tumor angiogenesis and proliferation by obesity and dyslipidemia^12, 26–29^.

Recently, thyroid dysfunction has been reported as a risk factor for certain cancers. However, the effect of THR, which may result in insufficient or excessive replacement, on the risk of secondary primary cancer has not been well elucidated. In this respect, our study, using big data based on the entire population of South Korea, supports the deleterious effect of THR on the risk of second primary cancer.

The American Thyroid Association (ATA) recommends 0.1 mU/L as the target TSH for full suppression in patients with high-risk DTC. However, long-term suppression of TSH, especially in the undetectable range, often increases the risk of cardiovascular events such as atrial fibrillation. In line with this idea, the ATA also recommends that TSH suppression be flexible over time in patients with low- or intermediate-risk DTC^30–33^. In this study, the patient group without THR had the lowest risk of second primary cancer. The group with a THR for > 5 years had the highest risk. Regarding the dose of thyroid hormones, patients with a high dose (> 100 μg/day) also have a high risk of second primary cancer. Considering the cardiovascular and metabolic adverse effects of long-term THR, clinicians may also need to address the risk of second primary cancer. Moreover, the risk of second primary cancer was also increased by short-term THR (≤ 1 year) and low doses (≤ 50 μg per day). In subgroup analysis for patients who underwent lobectomy, we noted that low doses could increase the risk of second primary cancer. The corresponding patients might have early discontinuation of THR therapy owing to a low risk of DTC recurrence. Based on this background, these patients might have had an insufficient replacement, although we could not present their thyroid hormone levels. Therefore, insufficient or excessive hormone replacement therapy might increase the risk of secondary primary cancer in patients with DTC.

Similarly, studies have shown that thyroid dysfunction increases the risk of developing cancer. A series of meta-studies have reported that thyroid dysfunction increases the risk of breast and prostate cancer^34^. A study of 20,990 patients with colorectal cancer in the UK reported that hyperthyroidism or untreated hypothyroidism increased the risk of developing colorectal cancer and that a long period of THR therapy decreased the risk^17^. The data of this study also showed that the risk of skin and melanoma was reduced in patients taking hormones for a long time compared to those who did not take hormones.

Furthermore, some studies have reported that thyroid hormones affect cancer cell proliferation^35, 36^. A Swedish population-based study reported that levothyroxine treatment increased the risk of all cancers, especially in women^18^. Previous studies have also presented results on the association of thyroid hormones with developing carcinomas, such as breast and lung cancer^35, 37–39^. Although the results of this study presented increased risks only in a small number of individual second primary cancers such as liver, lung, and brain cancers, combining these previous studies, the risks of second primary cancers were increased in the group of patients who took thyroid hormone compared to those who did not. The results of these studies and our study warrant further research on the occurrence of second primary cancer and the appropriate discontinuation period, in addition to the effects of supplemental factors of levothyroxine in patients with thyroid cancer. In some carcinomas, the risk of occurrence was higher in the group using levothyroxine for a certain period than in the group without levothyroxine; in certain carcinomas, the risk decreased regardless of the duration of use compared to the group without levothyroxine.

This study had several limitations. First, determining the scope of surgery after a thyroid cancer diagnosis is not sophisticated. Although the health insurance claim elements differed according to the scope of surgery, it was confirmed that they were not accurately reflected in the analysis process. Accordingly, the entire patient group was classified as the period of taking levothyroxine. If a more sophisticated surgical range could be set, a more accurate analysis of the effects of hormones in patients who underwent partial thyroidectomy would be possible. Second, actual patient thyroid function was not assessed. As the HIRA database did not contain laboratory results, we were unable to obtain blood test values ​​before and after surgery; therefore, we could not rules out the effects of other factors on the study results. Third, even though we used a nationwide database, the number of patients with second primary cancer was too small for the calculation of ORs for individual cancer types. Finally, even though we excluded the patients with a history of other cancers before surgery, as well as patients who died or developed a second primary cancer within 2 months after thyroidectomy, we could not definitely exclude existing (occult) multiple cancers.

In conclusion, insufficient or excessive hormone replacement therapy may increase the risk of secondary primary cancers in patients with DTC. Future studies with larger numbers of patients and more detailed variables are needed.

## Methods

### Data source

The data used in this study were extracted from the HIRA medical billing data. This comprehensive database collects all medical usage information registered by South Korea’s National Health Insurance Service. The HIRA database contains almost all the healthcare information for the entire South Korean population.

For assessment and repayment purposes, all medical records were integrated into HIRA claims data as electronic medical information. The database includes information about inpatient care, outpatient care, demographics, diagnoses, comorbidities, prescriptions, and surgeries. Thus, the HIRA database can obtain estimates representing the extent and severity of disease in an unselected population of patients. These data have been verified in many previous studies and are considered reliable^40–43^.

### Study population

Big data from the HIRA were used for analysis to secure a large study population. The HIRA database contains medical records and reflects the medical use and results of the entire Korean population. Data of adult patients aged between 19 and 80 years who underwent thyroid surgery at least once between January 2009 and June 2020 were extracted from this database. Patients who underwent thyroidectomy twice or more, those with a history of other cancers before surgery, and those who died or developed a second primary cancer within 2 months after thyroidectomy were excluded. Additionally, patients with no thyroid cancer diagnosis code before thyroid surgery or with a difference of ≥ 1 year between the initial thyroid cancer diagnosis and surgery were excluded. Data from patients with second primary cancers were extracted from the included patients. A patient with a second primary cancer was defined as having both a diagnosis and a cancer-exempted calculation code. Data of patients with second primary cancer and control patients matched for age, sex, operation date, and follow-up duration were extracted at a ratio of 1:4.

### Statistical analysis

Data are presented as the mean ± standard deviation for normally distributed continuous variables and as proportions for categorical variables. A nested case–control analysis was performed to exclude length bias and confirm the correlation between the duration of hormone drug administration, dose, and incidence of second primary cancer. Conditional logistic regression was performed to estimate the odds ratio (OR) and the corresponding 95% confidence interval (CI). Statistical significance was set at P < 0.05. SAS Enterprise Guide version 7.1 (SAS Institute Inc., Cary, NC, USA) was used for all the statistical analyses.

### Supplementary Information

Supplementary Tables.

## Data Availability

The datasets generated during and/or analysed during this study are available from the corresponding author on reasonable request.
