# Supplementary material for: Impact of thyroid hormone replacement on the risk of second cancer after thyroidectomy: a Korean National Cohort Study
Source: Sci Rep. 2023 Sep 28;13:16280. doi: 10.1038/s41598-023-43461-8 (PMC10539343; doi:10.1038/s41598-023-43461-8)
Supplement: Supplementary file 1 — Supplementary Tables. [file 41598_2023_43461_MOESM1_ESM.docx]

**Impact of thyroid hormone replacement on the risk of second cancer after thyroidectomy: A Korean National Cohort Study**

Joon Ho, MD^1†^, Minkyung Han, PhD^2†^, Inkyung Jung, PhD*^3^, Young Suk Jo MD, PhD*^4^, and Jandee Lee, MD, PhD*^1^

^1^Department of Surgery, Open NBI Convergence Technology Research Laboratory, Yonsei University College of Medicine, Seoul, South Korea

^2^Biostatistics Collaboration Unit, Department of Biomedical Systems Informatics, Yonsei

University College of Medicine, Seoul, South Korea

^3^Division of Biostatistics, Department of Biomedical Systems Informatics, Yonsei University College of Medicine, Seoul, South Korea

^4^Department of Internal Medicine, Yonsei University College of Medicine, Seoul, South Korea

^†^ The first two authors equally contributed to this work.

***Correspondence and requests for materials should be addressed to:**

[ijung@yuhs.ac](mailto:ijung@yuhs.ac) (Jung I.); [jandee@yuhs.ac](mailto:jandee@yuhs.ac) (Lee J.); [joys@yuhs.ac](mailto:joys@yuhs.ac) (Jo Y.S.) Tel: +82-2-2228-0752, Fax: +82-2-393-6884

This file includes Supplementary Table 1

**Supplementary Table 1. Baseline characteristics of patients with thyroid lobectomy according to secondary cancer in a Korean national cohort after matching**

|  | Second primary cancer | | P-value |
| --- | --- | --- | --- |
|  | Absence (n =8300) | Presence (n =2075) |  |
| Age at surgery, mean ± SD | 52.5 ± 11.7 | 52.5 ± 11.8 |  |
| ≤40 | 1100 (13.25) | 275 (13.25) |  |
| 40< and ≤60 | 4820 (58.07) | 1205 (58.07) |  |
| 60< | 2380 (28.67) | 595 (28.67) |  |
| Sex, n (%) |  |  |  |
| Men | 1592 (19.18) | 398 (19.18) |  |
| Women | 6708 (80.82) | 1677 (80.82) |  |
| Obesity, n (%) | 10 (0.12) | 6 (0.29) | 0.110 |
| Hypertension, n (%) | 2592 (31.23) | 716 (34.51) | 0.005 |
| Diabetes mellitus, n (%) | 1456 (17.54) | 407 (19.61) | 0.030 |
| Dyslipidemia, n (%) | 3423 (41.24) | 888 (42.80) | 0.208 |
| Infections*, n (%) | 461 (5.55) | 149 (7.18) | 0.006 |
| Duration of levothyroxine, n (%) |  |  |  |
| Mean ± SD (days) | 539.2±483.8 | 544.8±480.8 | 0.633 |
| No | 1517 (18.28) | 353 (17.01) | 0.532 |
| ≤1 year | 2174 (26.19) | 564 (27.18) |  |
| 1< and ≤5 years | 4599 (55.41) | 1156 (55.71) |  |
| 5 years < | 10 (0.12) | 2 (0.10) |  |
| Daily average dose of levothyroxine, n (%) |  |  |  |
| Mean ± SD | 82.8±56.2 | 84.2±57.9 | 0.321 |
| No | 1517 (18.28) | 353 (17.01) | 0.012 |
| ≤50 μg | 969 (11.67) | 291 (14.02) |  |
| 50 μg < and ≤100 μg | 3056 (36.82) | 725 (34.94) |  |
| 100 μg < | 2758 (33.23) | 706 (34.02) |  |

* Epstein-Barr virus (EBV), hepatitis B and C viruses (HBV and HCV), human immunodeficiency virus (HIV), human papilloma virus (HPV), human T-cell leukemia/lymphoma virus type-1(HTLV-1), and Helicobacter pylori (*H. pylori*)

**Supplementary Table 2. Multivariable analysis of the association between the daily average dose of thyroid hormone and the risk of second primary cancer in patients who underwent lobectomy**

| **Daily dosage**  **(avg. ㎍)** | 2^nd^ cancer | | **Unadjusted OR**  **(95% CI, P)** | **Adjusted OR**  **(95% CI, P)** |
| --- | --- | --- | --- | --- |
|  | Absence  no. (%) | Presence  no. (%) |  |  |
| No | 1517 (18.28) | 353 (17.01) | Ref. | Ref. |
| ≤50 | 969 (11.67) | 291 (14.02) | 1.30 (1.09–1.56, p = .004) | 1.31 (1.09–1.56, p = .004) |
| 50 < and ≤100 | 3056 (36.82) | 725 (34.94) | 1.02 (0.89–1.18, p = .780) | 1.03 (0.90–1.19, p = .652) |
| 100 < | 2758 (33.23) | 706 (34.02) | 1.11 (0.95–1.30, p = .202) | 1.13 (0.97–1.32, p = .130) |

Avg.: daily average, adjusted for obesity, hypertension, diabetes mellitus, dyslipidemia, and infections.; OR, odds ratio; CI, confidence interval.

**Supplementary Table 3. The impact of the duration of thyroid hormone replacement on the risk of individual second primary cancer**

| **Duration** | **Crude OR (95% CI)** | | | | | | |
| --- | --- | --- | --- | --- | --- | --- | --- |
|  | Lip & Tongue & Mouth | Stomach | Small intestine | Colon | Liver | Pancreas | Lung, bronchus |
| Second primary cases | 44 | 923 | 45 | 704 | 795 | 515 | 1622 |
| No | Reference | | | | | | |
| ≤ 1 years | 2.25  (0.23-21.94) | 1.31  (0.84-2.05) | 1.16  (0.10-12.82) | 1.16  (0.69-1.95) | 1.69  (1.12-2.55) | 1.34  (0.80-2.25) | 1.47  (1.07-2.02) |
| 1 < & ≤ 5 years | 2.20  (0.21-22.73) | 1.28  (0.87-1.88) | 1.82  (0.31-10.53) | 1.43  (0.92-2.25) | 1.16  (0.80-1.70) | 1.05  (0.67-1.66) | 1.40  (1.05-1.87) |
| 5 years 〈 | 4.04  (0.34-48.26) | 1.36  (0.87-2.12) | 3.47  (0.36-33.30) | 1.34  (0.78-2.30) | 1.02  (0.63-1.64) | 1.47  (0.81-2.68) | 1.27  (0.90-1.80) |

| **Duration** | **Crude OR (95% CI)** | | | | | | |
| --- | --- | --- | --- | --- | --- | --- | --- |
|  | Thymus, mediastinum, heart | Bone, joints  & soft tissue | Skin, Melanoma | Breast | Uterus | Ovary | Prostate |
| Second primary cases | 221 | 371 | 274 | 2455 | 229 | 264 | 332 |
| No | Reference | | | | | | |
| ≤ 1 years | 1.25  (0.56-2.77) | 1.03  (0.54-1.96) | 0.38  (0.18-0.82) | 0.81  (0.63-1.04) | 1.27  (0.57-2.84) | 1.10  (0.51-2.35) | 1.64  (0.84-3.20) |
| 1 < & ≤ 5 years | 1.99  (0.96-4.12) | 1.07  (0.60-1.89) | 0.38  (0.22-0.69) | 0.87  (0.71-1.07) | 1.27  (0.66-2.47) | 1.29  (0.67-2.51) | 1.24  (0.69-2.20) |
| 5 years 〈 | 2.13  (0.80-5.70) | 1.12  (0.54-2.33) | 0.37  (0.19-0.73) | 1.05  (0.82-1.34) | 0.97  (0.42-2.20) | 1.17  (0.52-2.67) | 1.45  (0.75-2.80) |

| **Duration** | **Crude OR (95% CI)** | | | | | |
| --- | --- | --- | --- | --- | --- | --- |
|  | Kidney | Brain, CNS | Lymphoma | Multiple myeloma | Leukemia | Others |
| Second primary cases | 373 | 785 | 215 | 74 | 168 | 1723 |
| No | Reference | | | | | |
| ≤ 1 years | 1.32 (0.68-2.56) | 1.68 (1.04-2.73) | 0.99 (0.42-2.36) | 1.26 (0.27-5.88) | 1.14 (0.32-4.06) | 1.30 (0.96-1.76) |
| 1 < & ≤ 5 years | 1.47 (0.84-2.59) | 1.35 (0.87-2.09) | 1.24 (0.60-2.56) | 1.18 (0.31-4.47) | 1.64 (0.59-4.56) | 1.03 (0.79-1.34) |
| 5 years 〈 | 1.56 (0.79-3.08) | 1.29 (0.73-2.30) | 1.12 (0.46-2.72) | 2.02 (0.46-8.86) | 2.68 (0.86-8.34) | 1.31 (0.93-1.84) |
